# Experiences of Mothers Facing the Prognosis of Their Children with Complex Congenital Heart Disease

**DOI:** 10.3390/ijerph17197134

**Published:** 2020-09-29

**Authors:** Sunhee Lee, Jeong-Ah Ahn

**Affiliations:** 1 College of Nursing, The Catholic University of Korea, Seoul 06591, Korea; shlee418@catholic.ac.kr; 2 College of Nursing, Research Institute of Nursing Science, Ajou University, Suwon 16499, Korea

**Keywords:** congenital heart defects, mothers, caregivers, emotions, qualitative research

## Abstract

Mothers of children with complex congenital heart disease face unique challenges and emotional burdens, while their children go through physical and psychological difficulties during disease progression. In this study, we aimed to explore the in-depth experiences and feelings of mothers facing the prognosis of their children with complex congenital heart disease that was surgically corrected. This is a descriptive qualitative study. We conducted semi-structured, face-to-face interviews with 12 mothers of children with complex congenital heart disease at a tertiary hospital in Seoul, Korea. The interview data were analyzed by content analysis. Participants were mothers aged between 40–58 years whose children were diagnosed with complex congenital heart disease which was surgically corrected. Based on the content analysis, the mothers’ experiences and feelings were categorized as immense suffering and adapting to a new life. Under the main categories, the concepts included feeling of abandonment, anxiety with potentially losing their children, having hope, seeking reassurance, being encouraged, and trying to embrace the situation. Mothers who cared for their children with complex congenital heart disease expressed emotions that changed sequentially alongside physical and psychosocial changes in the children. The results of this study are valuable for understanding the experiences and emotions of mothers facing the prognosis of their children with complex congenital heart disease in order to aid in the development of programs that support these mothers.

## 1. Introduction

Congenital heart disease (CHD) is the most common class of major congenital malformations in children. It has been detected in about 1.3 million children, and is noted in approximately 1% of total live births worldwide, although there are slight variations among reported incidences in many population-based studies [1]. Since 1953, when CHD was first treated successfully using cardiopulmonary bypass, early and accurate diagnosis and effective treatment for CHD have become standard approaches for the management of CHD [2]. With such advances, there has been a large increase in the survival rate of children with CHD [3]. More than 80% of newborns with CHD are expected to survive into adolescence or adulthood after proper surgery [4,5].

Having a child with CHD impacts the whole family, especially mothers who face unique challenges and yearn to make a better life for their children [6]. Although genetic factors may not be the only cause of CHD, it is provoking mothers who birthed their children to feel guilty and have emotional difficulties [7]. In addition, difficulties in the early stages of initial diagnosis and treatment, various procedures, multiple hospitalizations, and numerous medications contribute to the emotional burden of mothers with children who have complex CHD [8].

Children with CHD have diverse physical and psychological difficulties compared to healthy individuals [9]. These factors also make the mothers responsible for enduring or overcoming difficulties during the developmental process and overall life of their children with CHD [6]. In a recent systematic review of 44 qualitative studies to explore the perception and sentiments of pediatric and adolescent patients with CHD, patients described their experiences as coping with a new sense of normality, feeling powerless as their health deteriorates, struggling through long lasting medical ordeals, and finally establishing new routines [10]. Challenges were also reported in previous studies focusing on parental emotions and experiences of children with CHD. For instance, mothers of newborns with CHD cope with demanding emotional and hands-on work [11], and the stress and coping challenges of mothers with infants with CHD changed as surgery approached [12]. The mothers of children with CHD (aged 7 months to 14 years) commented on the burden of childcare and tension resulting from the disease and difficulties of the care process [13]. In addition, mothers of children with CHD (between 6 months and 4 years) described their appreciation of the healthcare team on initial diagnosis, their possible optimism surrounding medical outcomes, and feelings of general uncertainty concerning the child’s upcoming medical care [14].

Although various studies have investigated the experiences of children with CHD, relatively scarce information exists regarding the experiences and feelings of mothers faced with their child’s complex CHD, which would be a life-threatening and painful diagnosis for the child. Thus, we aimed to explore the in-depth experiences and feelings of mothers facing the prognosis of their children with complex CHD that was surgically corrected and to present the evidence for developing interventions to effectively support them.

## 2. Materials and Methods

### 2.1. Study Design and Participants

This article reports the findings of a qualitative study using data obtained through semi-structured interviews and analyzed by content analysis. Participants in this study included 12 mothers of children with complex CHD. The inclusion criteria were mothers: (1) having a son or daughter who was diagnosed with complex CHD and had undergone heart surgery; (2) visiting for regular hospital follow-ups; and (3) available to participate in the study with a signed informed consent form.

### 2.2. Data Collection

Data collection was conducted at the cardiovascular center of a tertiary hospital in Seoul, Korea from June to November 2016. The participants were selected through a purposive sampling method which is a non-random sampling method used in qualitative research. Face-to-face interviews, using semi-structured and open-ended guiding questions, were conducted to gather information on the personal experiences of mothers of children diagnosed with CHD. Interviews were conducted by one of the researchers who was not directly involved in the treatment process of the participants’ children and was trained in qualitative interviewing. The researcher had 10 years’ experience in conducting qualitative research. Interviews were conducted in comfortable places chosen by each mother (i.e., café or park located near the participants’ residence) and lasted 60–90 min. Examples of guiding questions are as follows: “How did you feel when you first found out about your child’s disease?”, “What does the word “CHD” mean to you?”, “What were some difficult emotions you felt while raising your child who was diagnosed with CHD?”, “What were some good experiences you had with your child?”, and “What else do you think I should know about your experiences as a mother?”. Moreover, we provided the participants an opportunity to elaborate on their response with more follow-up questions such as, “Could you tell me more about it?” or “How would you describe these feelings?” in order to obtain in-depth data. We continued to collect data until saturation of the information. Audio files were created to record communication between the interviewer and interviewee after obtaining permission from the participants. The researcher noted the key points during the interview. These were then transcribed in full-length.

### 2.3. Data Analysis

Data were analyzed using a content analysis approach. The researchers went through the process of abstraction (transcribed data, meaningful phrases, concepts, and categories) one by one. To extract the various meaningful phrases, two researchers read and compared transcribed data continuously. The concepts were developed from separating the differences and gathering the similarities of the extracted meaningful phrases through the discussion of the researchers. They developed the categories by comparing the differences and similarities of the concepts [15]. To ensure trustworthiness of the data, criteria of credibility, transferability, dependability, and confirmability were applied throughout the research process [16]. To obtain credibility, participants were interviewed in their natural environments to encourage a more open discussion. To establish transferability, the researchers interviewed participants until no new information or themes were observed in the data. To ensure dependability of data, the researchers aimed to verify that their findings were consistent by sharing it with the participants. Participants made sure that the extracted concepts were representative of their experiences and provided feedback to fill in any data gaps. To establish confirmability, the researchers continuously checked and rechecked the data throughout the study to address any potential biases that could alter the findings [17,18,19,20]. Data were analyzed in Korean, and the translation and back-translation of the data were performed by professional translators. The researchers confirmed that there were no discrepancies.

### 2.4. Ethical Considerations

This study was conducted in accordance with the standards and ethical criteria of the Declaration of Helsinki and approved by the Institutional Review Board of Yonsei University Medical Center in Seoul, Korea prior to initiating the study. The researcher explained the purpose of the study, assured data confidentiality, and informed the participants about the interview being recorded before starting. All participants voluntarily agreed to participate in the study, completed written informed consent forms, and were assured that their information would remain confidential. Participants also understood that they could refuse to participate at any time, and that their decision would not affect their children’s treatment. The participants were enrolled in a previous study published elsewhere [21].

In order to ensure confidentiality, participants were identified by “Participant” followed by a sequence number (1–12) according to the order of participation in the study.

## 3. Results

### 3.1. Characteristics of the Participants

The general characteristics of the participants are shown in Table 1. Twelve mothers of children diagnosed with complex CHD were included in the study. The age range of participants was 40–58 years (mean 49.1 ± 5.4 years). Regarding the children, they were diagnosed with double outlet right ventricle, pulmonary atresia, tetralogy of Fallot, transposition of the great vessels, or pulmonary stenosis, and all had undergone surgical correction. About 83.3% of mothers responded that they received positive support from their husband in child rearing (Table 1).

### 3.2. Experiences of Mothers Facing the Prognosis of Their Children with Complex CHD

The analysis of the experiences of mothers facing the prognosis of their children with complex CHD produced two main categories of immense suffering and adapting to a new life, and six concepts of feeling of abandonment, anxiety with potentially losing their children, having hope, seeking reassurance, being encouraged, and trying to embrace the situation (Table 2).

#### 3.2.1. Immense Suffering

##### Feeling of Abandonment

The mothers felt abandoned from the time of their child’s diagnosis. When they first heard about their child’s diagnosis and the need for heart surgery, the mothers expressed that they felt the world had abandoned them. They felt unfairly treated and even angry with the world.

One mother said: *“I felt like a floating island among many people. It was immensely hard”* (Participant two).

Another mother said: *“I blamed and cursed heaven and the world, who did this to me… then I was at loss for words for a while”* (Participant six).

And another said: *“I thought to myself that I have lived a good life until then, but why me? Those who were not as good as I had healthy children, but why only me?”* (Participant eight).

And another said: *“Such an unfortunate affliction only came to me, and I felt resentment towards the world”* (Participant nine).

##### Anxiety with Potentially Losing Their Children

In this study, the mothers felt deeply frustrated by the anxiety or concern for the uncertain future including the thought that their child may die. In particular, when coping with tasks and circumstances beyond their limits, they even considered ending their life due to the immense frustration.

One mother said: *“My child had shortness of breath and difficulties all the time. I went back and forth to the hospital so many times. I had always been so frustrated with the anxiety of not knowing when my child might die”* (Participant one).

Another mother said: *“I just wanted to get rid of the two of us, the child and me, from the world. I thought about it again and again and put her in my car then drove out fast in tears”* (Participant nine).

Then another said: *“It was too hard for me. At that time, I even thought about dying with my kid who might die anyway”* (Participant 12).

#### 3.2.2. Adapting to a New Life

##### Having Hope

The mothers were able to stand up again and become hopeful, especially after their children’s heart surgery. The positive surgical outcomes and improvements in the conditions doubled their hopes for their children.

One mother said: *“How happy I was to see her alive and we were able to live together again after the surgery. I thought it would be a blessing if the child could live just like this”* (Participant six).

Another mother said: *“I could only see my child’s lips. They became really rosy after he got the surgery. I was really thankful to see them and very hopeful then”* (Participant seven).

##### Seeking Reassurance

The mothers tried to dispel anxiety by searching for information about other things in the future as a coping mechanism to deal with the current situation. They worried about whether the children could go to school, get a job, or build a family, among other things, and tried to find information for the future.

One mother said: *“Even though he survived after surgery, I soon became very worried about the future. He is handicapped. It is impossible for him to be self-reliant. Therefore, I searched the internet. The pamphlets at the hospital also provided a great deal of information. I read them all”* (Participant seven).

Another mother said: *“I always think about what he can do by himself in the future. I look for success stories of children with the same disease now becoming adults. Sometimes they come to the hospital and speak about their past experiences”* (Participant eight).

##### Being Encouraged

The mothers gained their strength again by rebuilding their courage from the surrounding environment. They were encouraged by other mothers and children they met in the hospital, by healthcare providers, and even by their own sick children.

One mother said: *“In the hospital, I met many mothers and their children who were in the same boat, with the same pain and grief, even though we were very different from each other. We all comforted one another. I really got a lot of courage to stick with my child from them”* (Participant two).

Another mother said: *“My child seemed to learn how to grow up. Even though she was very small, she said that she would take care of it. We finally leaned on each other”* (Participant five).

##### Trying to Embrace the Situation

Eventually, the mothers became emboldened and accepted their children’s illness and the situation as they were. They decided not to avoid their own children as well as the disease.

One mother said: *“I decided to think that I was going on a vacation with my child during our lives. We do not need to have a big one, just have fun in every moment”* (Participant two).

Another mother said: *“There is no way to avoid the given fate. I guess I just have to accept it. If I go to the hospital with the child and go after it hard, I believe it could get better someday”* (Participant five).

## 4. Discussion

The mother of a child with complex CHD is expected to be exposed to more diverse and specific experiences than the mother of a normal child. However, healthcare providers have less of an interest in attending to the emotional needs of mothers with children that have CHD, as most of the literature focuses on the experiences and needs of the sick child. The ongoing medical follow-ups for children, unexpected symptom changes, various cardiac procedures and surgeries, repeated hospitalization and discharge, and economic difficulty due to hospital expenses, all together put a heavy burden on mothers of children with CHD [22].

In this study, mothers who cared for their children with complex CHD expressed emotions that changed with the child’s disease progression. According to the findings, we derived two categories: immense suffering and adapting to a new life. Moreover, six conceptual components of the two categories were defined including feeling of abandonment, anxiety with potentially losing their children, having hope, seeking reassurance, being encouraged, and trying to embrace the situation.

In this study, the mothers expressed their emotions as undergoing great difficulty and feelings of unfair treatment and abandonment by the world at the moment of their child’s diagnosis, but they could also see the possibility and hope for a new life while looking at their children who were undergoing surgery and recovering. Similarly, a previous study showed that mothers of a child with CHD experienced various emotions and found themselves on a roller coaster of feelings from anxiety, shock, and fear to feelings of being blessed and relieved [23]. Mothers who lived with their sick children were forced to deal with fluctuating emotions depending on the child’s symptoms and state of post-heart surgery. Their disposition could be negative or positive; however, it was largely dependent on the child’s physical and emotional progress.

From the time of the child’s diagnosis of complex CHD, the participants in this study expressed feelings of abandonment and anxiety with potentially losing their children. It is demonstrably difficult process for mothers who care for children with complex CHD, and such hardships have been reported in various studies. For instance, one study reported that mothers of children with CHD suffered from enormous tension, difficulties, and bitter realities in the rearing of their sick children [13]. In addition, another study found that mothers of children with CHD felt a complex combination of negative emotions such as anxiety, worry, fear, guilt, hopelessness, and helplessness [24]. A recent systematic review about parents of a child with CHD revealed that about half of the parents expressed elevated depressive symptoms, and up to 80% reported psychological distress [22]. Furthermore, it has been reported that mothers of children with CHD have significantly lower quality of life regardless of the severity of the children’s disease [25]. When the patient’s symptoms were out of control or worsened, we found that the mothers experienced a similar hardship as the child in this study. Therefore, it is necessary for healthcare providers to develop early screening methods for the psychological distress of mothers of children with CHD, and feasible psychosocial interventions for them should be considered as an important part of the comprehensive care for CHD patients from early diagnosis.

Although mothers generally experience negative emotions regarding their child’s diagnosis, they also find ways to cope and manage their stress. For instance, according to this study, mothers experienced positive feelings of hope after their children underwent surgery, since the surgery alleviated their symptoms. Similarly, a previous study showed that mothers described the experience of joy and gratitude when they were finally able to see their children again after surgery [23]. On another note and as demonstrated in this study, even in times of uncertainty of their children’s future, mothers often tried to acquire knowledge and success stories through the internet or hospital resources of children with the same disease. This search for information provided mothers with reassurance and sense that they were not alone in the fight. This same theme has also been observed in previous studies in which mothers of children with CHD frequently tended to absorb an overwhelming amount of information in seek of reassurance and hope [23].

In this study, after a certain post-surgery period, the mothers began feeling encouraged and tried to embrace the situation. They said that they gained their strength by meeting other mothers in similar positions, supportive family members, healthcare providers, or even having their sick children always by their side. Similar to this, a previous study revealed that about 80% of parents of children with CHD showed moderately helpful coping strategies that regulated their stress, even while they were experiencing a great burden due to their child’s disease [26]. Such positive coping strategies can contribute towards maintaining the family unit of children with CHD [27]. Another study suggested that mothers of children with CHD need to be supported and encouraged to practice self-care in order to appropriately care for their children [28]. Therefore, it is important for healthcare providers to be aware of the need for care of mothers of children with CHD, and to pay attention to the medical and social support that can better facilitate a positive emotional state in mothers [27]. These efforts are necessary not only for mothers but also for CHD patients, their families, and communities.

Mothers of children with complex CHD experience diverse emotions of varying intensities as the child’s disease progresses. Instead of considering these feelings as natural or just avoiding them, healthcare providers should fully understand the mother’s emotions and expressions in accordance with the disease and treatment progress. Furthermore, it should be considered that the negative experiences of mothers are not all encompassing and could be channeled in a positive direction with timely support. In addition, it needs to be acknowledged that these complications are not only experienced by sick children but also by their mothers. Therefore, supportive intervention programs need to be developed and provided for mothers who spend most of their lifetime taking care of their children with complex CHD.

This study has several limitations. First, we included only mothers who regularly visited the hospital and did not include those who did not follow the hospital’s regular program, which can affect their experiences and feelings. Second, this study included only mothers of children with CHD who were surgically treated and did not include mothers of children who had not commenced treatment. There may be a difference in emotions and experiences between mothers of children who have and have not received treatment yet. Third, a small sample is not representative of the entire population and therefore the results are not generalizable. Fourth, the interviews were not conducted immediately after diagnosis or surgery and were based on the mothers’ recollections. Further research is necessary to verify and compare the findings in various settings with a larger population.

## 5. Conclusions

Mothers facing the prognosis of their children with complex CHD expressed diverse experiences, which varied sequentially in accordance with the child’s disease progression. The experiences and emotions of mothers who provide lifelong care for their children will continuously change along with the children’s physical and psychological alterations.

Healthcare providers need to understand these experiences and build family-centered education and supportive interventions to better advocate for mothers and facilitate better treatment for both children with CHD and their mothers.

## Figures and Tables

**Table 1:** General characteristics of the mothers and their children with complex congenital heart disease.

Mother	Age	Gender of Child	Cause of the Surgery of Child	Husband’s Support in Child Rearing
1	40	M	DORV	No
2	48	M	TOF	Yes
3	53	M	DORV	Yes
4	54	F	DORV	Yes
5	50	F	PA	Yes
6	58	F	DORV	Yes
7	43	M	TOF	Yes
8	57	M	PA	Yes
9	49	F	TGV	Yes
10	46	M	PA	No
11	47	M	PA	Yes
12	45	F	PS	Yes

DORV = double outlet right ventricle, TOF = tetralogy of Fallot, PA = pulmonary atresia, TGV = transposition of the great vessels, and PS = pulmonary stenosis.

**Table 2:** Categories and concepts of the experiences of mothers of children with complex congenital heart disease.

Categories	Concepts
Immense suffering	Feeling of abandonment
	Anxiety with potentially losing their children
Adapting to a new life	Having hope
	Seeking reassurance
	Being encouraged
	Trying to embrace the situation
